# Synchronic nasopharyngeal and intraparotid warthin tumors: A case report and literature review

**DOI:** 10.4317/jced.51441

**Published:** 2014-10-01

**Authors:** Karina-Lizbeth Yáñez-Barraza, Hugo-Ricardo Domínguez-Malagon, Adalberto Mosqueda-Taylor, Ana-María Cano-Valdez, Kuauhyama Luna-Ortiz

**Affiliations:** 1 Department of Health Care, Universidad Autónoma Metropolitana Xochimilco. Mexico City; 2 Department of Surgical Pathology, Instituto Nacional de Cancerologia. Mexico City; 3 Department of Head and Neck Surgery, Instituto Nacional de Cancerologia. México City

## Abstract

Warthin tumor is the second most frequent benign salivary gland tumor after pleomorphic adenoma; it occurs almost exclusively in the parotid gland and peri-parotideal lymph nodes, although it may rarely present in other locations. It may be multicentric and bilateral in a small percentage of cases. Nasopharyngeal Warthin tumor is very rare, and the presence of a synchronic WT involving nasopharynx and parotid is an exceptional event, as it has been described only twice in the literature. In this article we report an additional case of a synchronic Warthin tumor and review the related literature.

** Key words:**Warthin tumor, synchronic WT, multicéntrico, nasopharynx.

## Introduction

Warthin tumor (WT) was described for the first time in 1895 by Hildebrand as a variant of lateral cervical cyst (1) and 15 years later it received the name of Papillary Cystadenoma in a publication done by Albrecht and Arzt (2). In 1929 Warthin reported two similar cases and described the lesion as a slow-growing benign lymphoepithelial neoplasm, applying the name Lymphomatous Papillary Cistadenoma (3).

 Warthin tumor is the second most frequent benign salivary gland tumor after pleomorphic adenoma; it occurs almost exclusively in the parotid gland and peri-parotideal lymph nodes, it may be multicentric in up to 12-20%, and bilateral in 5-14% of cases (4). Although extraparotideal WT is rare, isolated cases involving oral cavity (5), larynx (6) and cervical lymph nodes have been described (7). Nasopharyngeal WT is very rare, up to date only 5 cases in this location have been reported (8-12). However the presence of a synchronic WT involving nasopharynx and parotid is an exceptional event, and it has been described only twice in the literature (13,14). Histogenesis of extraparotideal WT is controversial, several hypotheses have been proposed, the most accepted one suggests that it is due to delayed encapsulation of the parotid gland during the embryologic development, a theory that supposes some ducts and acini become trapped in extraparotideal lymph nodes giving rise to the tumor (9,12,15). In the present article we report another case of a synchronic tumor and review the related literature.

## Case Report

**Figure 1:**
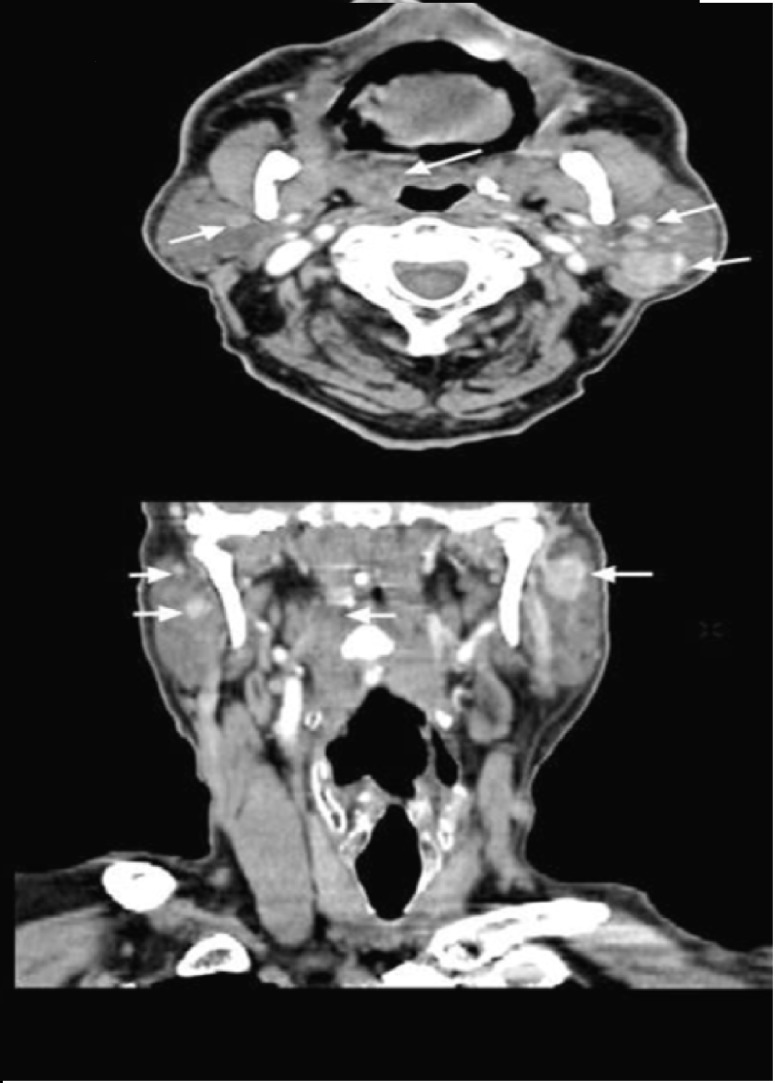
CT scan with endovenous contrast and multiplanar reconstruction: Hyperdense lesions are seen in both parotid glands, two in each gland are identified, all lesion have well-defined borders, the largest one measures 27 x 19 mm. There is asymmetry of the Waldeyer’s ring with increased volume and hyperdensity of the right side, and contralateral calcifications.

A 77 year-old man developed a slightly painful mass in the left side of the neck and weight lost of 2 kg over the last three months. In the past he worked in a recycling plant where he had direct contact with lead compounds for two months. The patient was a smoker for 49 years with a daily consumption of 18 cigarettes, and occasionally drank alcohol. Ten years before he had a myocardial infarct and was treated with antihypertensive drugs for the last five years (metoprolol). He is also being controlled for Diabetes Mellitus with oral drugs for the last 5 years. On physical examination a 6 x 3 cm tumor was found in the left parotid gland. A nasal endoscopy disclosed a poorly delimited tumor in the Rosenmüller fossa measuring approximately 1x1 cm. Presence of these lesions was confirmed by computed tomography (Fig. 1). A superficial parotidectomy and biopsy of the nasopharyngeal lesion were performed.

## Pathological Findings

**Figure 2:**
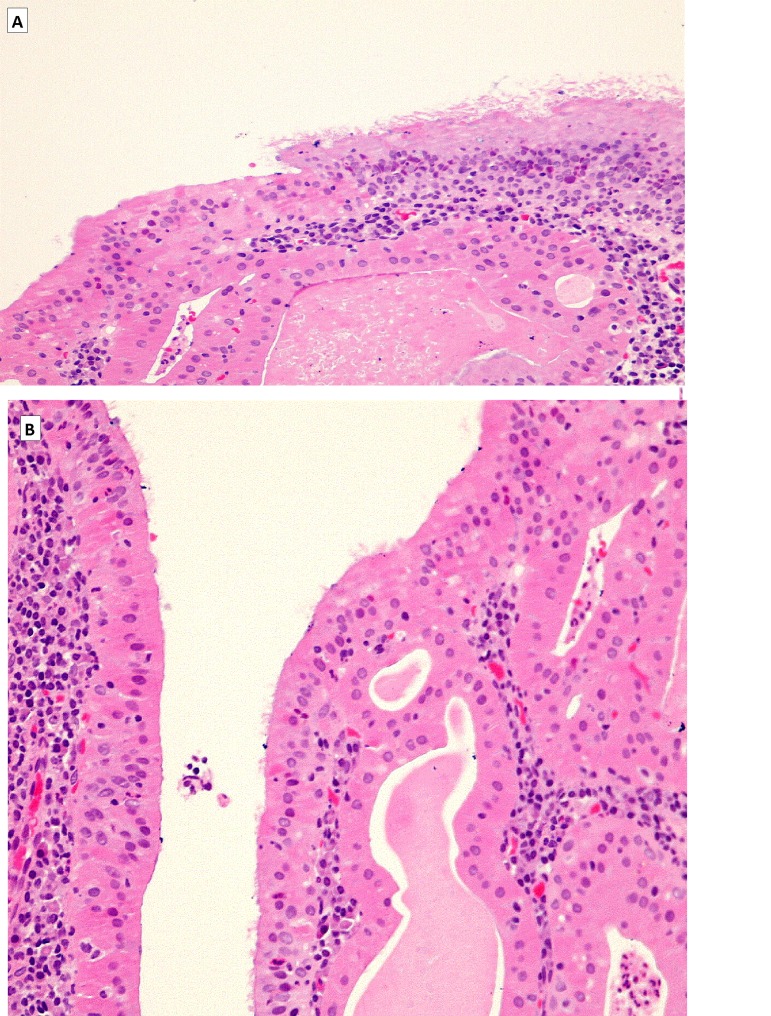
(A) Low power view of nasopharyngeal tumor: Adenoid tissue with glandular and follicular structures lined by cubical cells with ample eosinophilic cytoplasm. Follicular lumens contains proteinaceous material. (B) High power view show on the surface ciliated epithelium partly replaced by cubical oncocytic cells with bland nuclei.

The nasopharyngeal tumor on gross inspection consisted of several fragments of bland, pale pink soft tissue measuring up to 3 mm. Histologically it was characterized by papillary projections, and the surface was partially covered with ciliated epithelium. The papillae were lined by columnar cells with ample eosinophilic, granular cytoplasm, the nuclei were uniformly oval, with bland chromatin and inconspicuous nucleoli, and no mitotic activity was detected. The stalks of the papillae and adjacent connective tissue contained mature lymphoid tissue with a dense population of lymphocytes and plasma cells (Fig. 2).

**Figure 3:**
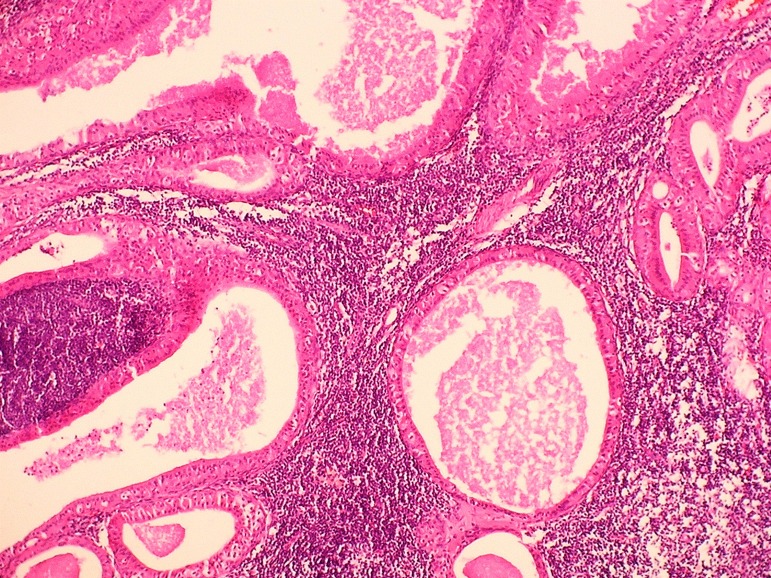
Low power view of parotid tumor: Multiple cystic structures lined by cubical oncocytic cells. The supporting stroma shows lymphocytic infiltrate and formation of lymphoid follicles.

On gross examination, the superficial lobule of the parotid gland measured 7.5 x 3.5 x 2.5 cm, showing on cut section two well circumscribed nodules, the largest measured 4 x 2 x 1.5 cm. The cut surface had a granular appearance with dark brown areas alternating with light brown zones. Histologically both nodules were well delimited and showed cystic and papillary structures delineated by columnar cells with bland nuclei and abundant eosinophilic granular cytoplasm; the stoma displayed copious lymphoid tissue. The histological features were similar to the nasopharyngeal tumor (Fig. 3). Immunohistochemical studies were done to confirm the reactive nature of the lesion and showed: Epstein-Barr virus (LMP) was negative. Most of the lymphoid cells were B (CD20+). CD3+ T cells constituted less 25% of the population with a CD4:CD8 ratio of 4:1. Five years after the surgical treatment the patient is asymptomatic, with no evidence of relapse.

## Discussion

Warthin tumor arises almost exclusively in the parotid gland, it frequently is associated to smoking, as the risk for developing it is 8 times higher in smokers than in non-smokers (15), our patient had a history of intense smoking for almost 50 years. Nasopharyngeal location of WT is a rare event, with only 5 cases informed to date (8–12). The synchronic presentation of WT in nasopharynx and parotid gland is exceptional. The first case was reported by Low *et al*. in 2002 (13), the patient was a 53 year-old Chinese woman with a 3 cm nodule in the right side of the neck behind the mandibular angle, and a naso-endoscopy showed a 1 cm nodule located in the postnasal space. The histological examination rendered the diagnosis of WT in both locations. Hilton et al. reported in 2008 the case of a 55 year-old man with multifocal WT that affected both parotid glands, post-nasal space, base of the tongue and tonsils (14).

Based on the literature review ( Table 1), nasopharyngeal WT has a predilection for old individuals, with a mean age of 69.5 years (range 53-81 years), and there have been 5 male and 3 female patients. Heavy smoking was reported in three patients and is not mentioned in the remaining five. Most tumors were small, four measured less than 1 cm, three were 2 to 3 cm in diameter, and size was not mentioned in one. Symptoms were related to their nasopharyngeal location near the Eustachian tube, and included: otalgia, cough, dizziness, hypoacusia, and nasopharyngeal discomfort. The time to diagnosis was 3 to 24 months with a mean of 14 months.

**Table 1:**
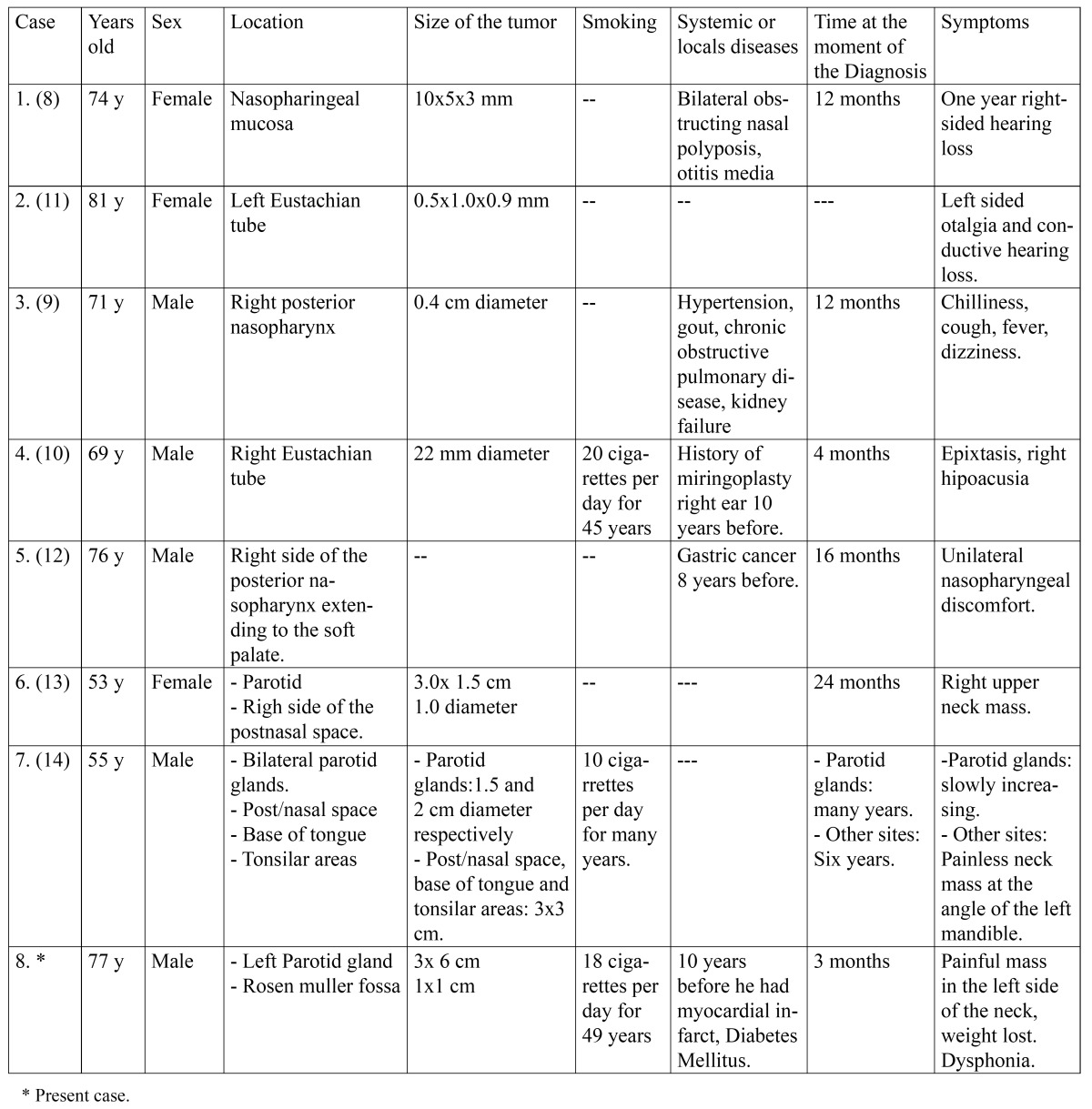
Nasopharyngeal Warthin Tumors reported in the literature.

Histogenesis of extraparotideal WT has been widely debated and actually is controversial. Several hypotheses have been proposed, the most accepted one suggests that it is due to delayed encapsulation of the parotid gland during the embryologic development, a theory that supposes some ducts and acini become trapped in extraparotideal lymph nodes giving rise to the tumor (9,12,15).

Regarding the lymphoid component, some authors consider it as a pre-existing lymph node trapped in the gland, and the lymphoid response represents an exaggerated immune response (9). According to the so-called heterotopic theory it is possible that during the embryonic development, epithelial cells from the second, third and fourth pharyngeal pouches that normally descend to the mediastinum become trapped in lymphoid organs; this could explain the extraparotideal location of WT (16). However, the epithelium of the pharyngeal pouches is dissimilar to the epithelial component of WT. According with this theory Seifert *et al*. (17), classified WTs into different subtypes based mainly on their lymphoid component, these authors proposed a staged pathogenic development in which subtype III (lymphoid predominance) WT constitutes an initial stage from which the subtype-I (typical) and subtype II (Epithelial predominance) WTs subsequently evolve. However, the amount of lymphoid tissue often exceeds that of the small lymph node from which it supposedly originates (16).

Other hypothesis postulate that in pharyngeal WT the lymphoid cells are reactive, polyclonal, and have a B-cell predominance, this chronic inflammation would induce oncocytic metaplasia of salivary epithelium trapped in lymphoid tissue (9). Other authors suggest that the lymphoid component of nasopharyngeal WT could represent an immune response (11,16). Some authors propose that virus infect ductal epithelial cells, and the release of gene products or cytokines by infected cells may activate lymphoid tissue and result in a polyclonal B/cell response (18-20). On this regard, some authors have suggested as a cause chronic obstruction of the nasopharinx (8) and intense smoking as an irritating factor (10,14), the lymphoid population in our case was polyclonal with B cell predominance indicating a reactive phenomenon as suggested by Yeh *et al*. (9).

Although the presence of synchronic tumors in the same patient may be coincidental, it is important to try to establish a link between them. WT can occur synchronously with other neoplasias such as pleomorphic adenoma Seifert *et al*. (21) and Low *et al*. suggested that in their case the parotid WT could be metastasis from the nasopharyngeal WT, a dubious explanation because both tumors were histologically benign (13). However, some tumors with bland histology, such as ameloblastoma and pleomorphic adenoma, may produce metastasis after many recurrences, which can still preserve benign histomorphological features (22,23).

Other authors postulate that multiple systemic factors such as nutritional or metabolic deficiencies, genetic factors, environmental influences, duct obstruction or chronic inflammation may concur to generate oncocytic metaplasia that could be the initial stage of a synchronous development of WT (11). Yeh *et al*. believes that the chronic inflammation in the nasopharynx induces the formation of oncocytic metaplasia of the glandular tissues in the stroma (9).

In conclusion, simultaneous WT in the nasopharynx and parotid gland has been reported only twice before, and although this occurrence may be coincidental, a common pathogenesis or a metastatic mechanism have to be considered. Even though the number of cases of extraparotideal WT is small, further investigation is required to establish if there are clinical, demographic and pathological differences among patients with intraparotideal, extraparotideal and synchronic WT.
